# Sociodemographic Status, Dietary Intake, and Physical Activity in Relation to Body Mass Index Among Female Adolescents in Saudi Arabia

**DOI:** 10.3390/children12070823

**Published:** 2025-06-22

**Authors:** Leila Arfaoui, Afnan Alghanmi, Ruba Alamri, Nouf Aljehani, Areej Alkhaldy, Mourad Assidi

**Affiliations:** 1 Clinical Nutrition Department, Faculty of Applied Medical Sciences, King Abdulaziz University, P.O. Box 80324, Jeddah 21589, Saudi Arabia; aalghanmi@kau.edu.sa (A.A.); riaamari@stu.kau.edu.sa (R.A.); naljehani0038@stu.kau.edu.sa (N.A.); aalkhaldy@kau.edu.sa (A.A.); 2 Institute of Genomic Medicine Sciences, King Abdulaziz University, Jeddah 21589, Saudi Arabia; massidi@kau.edu.sa

**Keywords:** BMI, sociodemographic status, dietary habits, physical activity, adolescents, obesity, Saudi Arabia

## Abstract

Background: Sociodemographic status, dietary intake, and physical activity have been linked to body mass index (BMI) among adolescents. However, there is a scarcity of research investigating these factors in relation to BMI standard deviation score (BMI_SDS_) in Saudi Arabia. Therefore, we examined the roles of sociodemographic status, dietary habits, and physical activity in relation to body mass index among Saudi female adolescents aged 11–18 years attending public and private schools in the city of Jeddah. Methods: This school-based cross-sectional study was performed between February and April 2019 in Jeddah. A total of 920 female adolescent students were recruited from both public and private schools. Data was collected using questionnaires conducted via interview as well as anthropometric measurements. Results: About 37.4% (*n* = 344) of the participants were overweight or obese. The majority of the participants (61.6%, *n* = 567) had a healthy dietary intake score. More than half of the participants (52.6%, *n* = 484) had a low physical activity and screen time score, with 36.5% (*n* = 336) never engaging in ≥60 min of physical activity per day and 61.4% (*n* = 565) reporting a daily screen time of >4 h. Multivariate logistic regression analysis revealed that students aged over 16 years were less likely to have overweight/obesity compared to those aged <14 years (adjusted odds ratio “aOR” = 0.53; 95% CI: 0.35–0.79, *p* = 0.002). Participants enrolled in private schools were at higher risk of being overweight/obese compared to those enrolled in public schools (aOR = 1.55; 95% CI: 1.16–2.08, *p* = 0.003). Adolescent females with daily vegetable intake were less likely than those who never consumed vegetables to be overweight or obese (aOR = 0.47; 95% CI: 0.29–0.77, *p* = 0.002). However, no associations were found with the other sociodemographic, dietary intake, physical activity, and screen time exposure variables. Conclusions: This study shows a trend of elevated obesity prevalence among Saudi female adolescents in Jeddah, highlighting the need for gender-sensitive, school- and community-based interventions targeting diet, physical activity, and sedentary behavior. To obtain a more comprehensive understanding, studies involving nationally representative samples, encompassing all cities across Saudi Arabia and exploring broader aspects of nutrition and physical activity, are essential.

## 1. Introduction

Adolescence is a sensitive period of time, especially among females, when biophysiological changes are occurring and dietary habits and activity levels are transforming [1,2,3]. The prevalence of overweight and obesity in children and adolescents is rapidly increasing worldwide, which has been recognized as a global public health concern [4]. In fact, adolescent obesity is strongly correlated with an increased risk of obesity in adulthood and is associated with a broad array of adverse health outcomes, including adult diabetes, cardiovascular hypertension, dyslipidemia, fatty liver disease, and premature mortality [5,6,7]. In addition, adolescent obesity can lead to psychosocial complications, such as a negative self-image, low self-esteem, and depression, and is also reported to have an influence on gynecological health during adulthood [8,9].

Saudi Arabia is characterized by a young population where 71% of the Saudis are below 35 years, and more than 27% of them are aged between 14 and 19 years [10], and hence deserve further focus as they are a potential growth and development in the future. However, the globalization and the adoption of Western lifestyles and modernization over the last few decades have markedly impacted Saudi society and contributed to increased obesity among adolescents [11]. It is also important to highlight that a systematic review of 51 studies involving *n* = 97,666 adolescents revealed that between 2002 and 2010, the prevalences of overweight and obesity among Saudi female adolescents increased from 17.4% to 27.4% and from 7.2% to 10.0%, respectively [12].

Sociodemographic status, dietary intake, and physical activity are among the main factors reported to play major roles in the prevalence of obesity among children and adolescents both globally and in Saudi Arabia [13,14,15]. In fact, unhealthy dietary habits characterized by high intake of energy-dense foods containing sugar, salt, and saturated fat, such as fast food, sweets, and sugary beverages, alongside nutrient-poor foods, are contributing factors to obesity among Saudi children and adolescents [16,17]. Moreover, physical inactivity and prolonged screen time exposure were also reported to be significant contributors to obesity. Low physical activity and sedentary lifestyles like long-term screen exposure are independently associated with obesity and metabolic risk [18,19,20]. Additionally, socioeconomic factors, such as income, parents’ education and occupation, and type of school, were reported to be associated with children/adolescents’ BMI [21,22]. Al-Hazzaa and collaborators in their Arab Teens Lifestyle Study (ATLS) initiative reports have clearly highlighted this multifactorial aspect of adolescent obesity [23]. Tackling all these factors early in youth life is crucial because they may remain the same into the childbearing years, making it more challenging to modify them later in life [24]. Although genetics is also considered one of the factors contributing to obesity among adolescents, suboptimal lifestyle choices, including unhealthy dietary intake and a lack of physical activity, are notably more significant and can be modified [11].

In the current study, the authors focused particularly on Jeddah city as the second largest and one of the most modern cities in western Saudi Arabia. In fact, Jeddah city has been undergoing radical changes in a rapidly emerging economy, accompanied by noticeable unhealthy lifestyle transitions. We thus considered it of particular interest to perform an updated and integrative analysis of the sociodemographic status, dietary habits, physical activity, and screen time activity in relation to body mass index among Saudi female adolescents aged 11–18 years attending public and private schools in Jeddah. The focus on female adolescents in this study is driven by reports showing elevated glucose levels and increased waist circumference (abdominal obesity) among female adolescents [25,26], highlighting a significant health concern [26]. Furthermore, Al-Nuaim (2022) revealed that 73% of Saudi female adolescents are physically inactive, compared to only 32% of their male counterparts [27], with 91% of females reported spending more than 2 h per day on screen time compared to 84% of male adolescents [28].

These particularities of Jeddah city should also be considered in the national context. In fact, and besides her demographical and socioeconomic context as a main country in the MENA region, Saudi Arabia is currently engaged in several transformative socioeconomic initiatives driven by the Saudi Vision 2030 towards the enhancement of the quality of life, the promotion of physically active lifestyles, the extension of health span, and the Saudi women empowerment [29,30]. Moreover, the prevention of chronic diseases as obesity, is among the top national research, development, and innovation priorities [31].

Therefore, the aim of this unique study is to explore children and adolescent obesity as a major public health challenge in the particular city of Jeddah (with dominant westernized lifestyles) affecting female youth using validated tools through combining multiple lifestyle and sociodemographic variables in a comprehensive approach (instead of scattered factors) to assess independent predictors of obesity among Saudi female adolescents in Jeddah city. Of note and compared to most previous studies done in the region, this study used the recommended corrected BMI Standard Deviation Score (BMI_SDS_) as a more accurate, reliable, age- and gender-corrected statistical measure adapted for evaluating growth and development in children and adolescents and planning suitable interventions [32,33].

The findings of this study are well aligned with the Saudi national priorities targeting the quality-of-life improvement and the women empowerment efforts and will help in understanding the factors influencing Saudi female adolescent lifestyles and obesity, and represent a reference knowledge baseline to suggest appropriate recommendations, including school-based approaches and guidelines to prevent obesity, uphold healthier lifestyles, and promote public health.

## 2. Materials and Methods

### 2.1. Study Design and Subjects

This cross-sectional study was conducted using a sample of female adolescent students aged between 11 and 18 years from middle and high schools in Jeddah, Saudi Arabia. The schools were selected using a multistage stratified random-sampling procedure, taking into consideration the school type, geographic area, and student grade. First, the schools were stratified into private and public schools. Three schools (two public and one private) were selected from each of the four geographic areas of the city (north, east, south, and west), giving a total of 12 schools. Classes were then randomly selected from each of the seven grades (grade 6 to grade 12) in each school. The total sample set consisted of 920 adolescents. Data was collected between February and April 2019. Students suffering from any chronic disease, such as diabetes, hypertension, kidney disease, or anemia, were excluded. Ethical approval was obtained from the Research Ethics Committee at King Abdulaziz University Hospital (ref. no: 72-19). Permission to collect data in schools was obtained from the director of the School Health Affairs Department, Ministry of Education, Saudi Arabia. Prior consent for participation was obtained from the adolescents and their parents. The questionnaires were collected and transmitted anonymously.

### 2.2. Procedures and Data Collection

The data were collected by trained clinical nutrition students via interview using a paper-pencil approach with manual data entry. This questionnaire was developed based on two previous studies. The first section, which includes sociodemographic questions, was adapted from a Saudi study by Alazzeh et al. [15] to ensure relevance to the local context. The sections that were focusing on dietary habits, physical activity, and screen time were based on the Canadian study by Janssen et al. [34]. The questionnaire was initially created in English, then translated from English to Arabic using the Brislin backtranslation method. The questionnaire underwent face validation by sharing the questionnaire with three PhD holders in nutrition and one dietitian. The questionnaire was then modified based on their responses. In addition, the questionnaire was pretested to ensure clarity and relevance before distribution to students and their parents. To assess the reliability of the questionnaire, a reliability analysis was conducted using Cronbach’s Alpha. The results showed a Cronbach’s Alpha value of 0.710, indicating acceptable internal consistency, while the validity test yielded a value of 0.851, reflecting strong validity for the questionnaire.

The questionnaire consisted of 23 questions split into four sections, including personal information (age, BMI, family medical history, school region, and living status). Anthropometric measurements, including body weight and height, were performed by trained clinical nutrition students following a standardized procedure [35]. A calibrated portable digital scale was used to measure body weight in kilograms to the nearest 0.1 kg. For accurate measurements, participants were weighed in light clothing without shoes, bags, or any head accessories. Height was measured to the nearest 0.1 cm with the head in the Frankfurt plane position and without shoes. The BMI standard deviation score (BMI_SDS_) was calculated according to World Health Organization (WHO) growth reference data for children aged 5–19 years [36] and was used to assess weight status relative to the study population. The categories were defined as follows: underweight (SDS < −2), normal weight (−2 ≤ SDS < +1), overweight (+1 ≤ SDS < +2), and obese (SDS ≥ +2) [36].

#### 2.2.1. Sociodemographic Status

The sociodemographic status (School type, family size, living status, house ownership, having a housemaid and/or driver, monthly parental income, and parental education level and occupation) was collected. Overall sociodemographic status was evaluated by summing the scores of the 10 questions and categorizing the resulting values as “most deprived” (scores of 0–7), “deprived” (8–15), “m” (16–23), or “affluent” (24–31).

#### 2.2.2. Dietary Intake Analysis

The dietary habits section consisted of seven questions. The participants were asked how many times per week they consumed each of the following six food items: fruits, vegetables, soft drinks, fast food, pastries or sweets, and potato chips. Each question had five possible responses: daily, 5–6 days per week, 2–4 days per week, ≤1 day per week, or never. The responses were scored from zero to four, with a higher score for healthier habits. The seventh question asked about the number of meals and snacks consumed per day. It had three possible responses (<3, 3–5, or >5), which were scored as one if the number of meals and snacks was between three and five and otherwise scored as zero. Overall dietary habits were evaluated by summing the scores of the seven questions and categorizing the resulting values as “unhealthy” (scores of 0–6), “somewhat healthy” (7–13), “healthy” (14–20), or “very healthy” (21–25).

#### 2.2.3. Physical Activity and Screen Time Assessment

The physical activity and screen time section contained two questions. In the first question, the participants were asked how many days per week they were physically active for 60 min or more. This question had five possible responses: daily, 5–6 days per week, 2–4 days per week, ≤1 day per week, or never. The responses were scored from zero to four in order of increasing frequency. In the second question, the participants were asked how many hours per day they watched or used a television/cellphone/tablet. This question had four possible responses: >4 h, 2–4 h, <2 h, or none. The responses were scored from zero to three in order of decreasing screen time. The physical activity and screen time scores were combined to evaluate the overall activity levels of the participants, where a higher overall score indicated healthier activity levels. The combined scores of the two questions were categorized as “low activity level” (scores of 0–2), “moderate activity level” (3 or 4), “good activity level” (5 or 6), or “high activity level” (7).

### 2.3. Statistical Methods

Statistical analysis was performed using SPSS Statistics version 28.0 for Windows (SPSS Inc., Chicago, IL, USA). Data were described using frequency statistics. Data were described using frequency statistics for categorical variables. Chi-square tests were adopted to explore sociodemographic variables, dietary habit scores, physical activity, and screen time scores associated with the BMI_SDS_ as a categorical variable. Binary logistic regression analysis was performed after exclusion of underweight subjects and considering overweight/obesity versus normal weight classification as dependent variables, and the significant variables from univariate analysis as independent variables. A *p* value of less than 0.05 was considered statistically significant.

## 3. Results

### 3.1. Participant Characteristics

Table 1 summarizes the characteristics of the study participants. A total of 920 female adolescents were recruited in this study. The participants were aged between 11 and 18 years, with a mean age of 15.1 ± 1.8 years. The mean BMI_SDS_ was within the normal range (22.7 ± 5.6 kg/m^2^). Among the participants, 59.0% had a normal BMI, 3.6% were underweight, 21.6% were overweight, and 15.8% were obese. In addition, 74.2% of participants reported a family history of chronic disease. The participants were distributed fairly evenly across the four geographic areas of Jeddah (27.5%, 21.2%, 21.5%, and 29.8% for north, east, south, and west, respectively). Most of the participants lived with both parents (84.2%), with the majority having a family size between five and seven members (65.4%) and living in their own house (67.1%). Almost half of the participants had a housemaid, a driver, and a high household income (50.1%, 51.0%, and 47.5%, respectively). Moreover, most of the participants had parents who had completed graduate studies (61.2% and 71.9% for the mother and father, respectively). With respect to parental occupation, most of the mothers were not working (55.7%), and slightly more than half of the fathers were working in a government position (50.2%) (Table 2).

### 3.2. Dietary Intake

Table 3 shows the dietary habits of the participants. Only 13.0% of the participants reported eating fruits on a daily basis, while 42.5% and 28.7% reported eating them ≤1 day per week and 2–4 days per week, respectively. Similar proportions of the participants (25.4–26.8%) reported eating vegetables ≤1 day per week, 2–4 days per week, and daily. Approximately half of the participants reported consuming soft drinks, fast food, potato chips, and pastries or sweets ≤1 day per week. In addition, approximately one-third of the participants reported eating fast food, potato chips, and pastries or sweets 2–4 days per week, and 25.5% reported that they never consumed soft drinks. In terms of the daily number of meals (including snacks), 48.2% and 44.6% of the participants reported consuming <3 meals per day and 3–5 meals per day, respectively, while only 7.3% reported consuming >5 meals per day. Overall, the dietary habit scores revealed that 30.2% and 61.6% of the participants had somewhat healthy and healthy dietary habits, respectively. In addition, 2.1% of the participants had unhealthy dietary habits, while 6.1% had very healthy dietary habits.

**Table 3:** Frequency of dietary intake of various food items, daily number of meals, and overall dietary intake scores (*n* = 920).

Food Item	Frequency *n* (%)				
	Daily	5–6 Days Per Week	2–4 Days Per Week	≤1 Day Per Week	Never
Fruits	120 (13.0)	54 (5.9)	264 (28.7)	391 (42.5)	91 (9.9)
Vegetables	234 (25.4)	101 (11.0)	237 (25.8)	247 (26.8)	101 (11.0)
Soft drinks	61 (6.6)	53 (5.8)	166 (18.0)	405 (44.0)	235 (25.5)
Fast food	51 (5.5)	82 (8.9)	318 (34.6)	437 (47.5)	32 (3.5)
Potato chips	51 (5.5)	77 (8.4)	273 (29.7)	427 (46.4)	92 (10.0)
Pastries or sweets	103 (11.2)	75 (8.2)	314 (34.1)	390 (42.4)	38 (4.1)
**Daily number of meals including snacks**	**<3**	**3–5**		**>5**	
	443 (48.2)	410 (44.6)		67 (7.3)	
**Dietary habit score** (total score = 25) *	**Unhealthy**	**Somewhat healthy**		**Healthy**	**Very healthy**
	19 (2.1)	278 (30.2)		567 (61.6)	56 (6.1)

Data are presented as numbers and percentages. * The dietary intake scores were categorized as “unhealthy” (scores of 0–6), “somewhat healthy” (7–13), “healthy” (14–20), or “very healthy” (21–25).

### 3.3. Physical Activity and Screen Time

Table 4 summarizes the physical activity and screen time reported by the participants. Overall, 52.6% and 32.3% of the participants displayed low and moderate physical activity and screen time scores, respectively. Good or high scores were recorded for only 15.1% of participants. In fact, 36.5% of the participants reported never being physically active for 60 min or more in one day during a typical week. Moreover, 33.9% reported being physically active for 60 min or more in a single day only once per week, and only 8.7% reported daily physical activity lasting at least 60 min. Furthermore, 61.4% of the participants reported >4 h of daily screen time, while 24.1% reported a daily screen time of 2–4 h.

### 3.4. Dependence of BMI_SDS_ Values on Sociodemographic Characteristics, Dietary Intake, Physical Activity, and Screen Time

Table 5 presents the association between sociodemographic factors, dietary intake factors, and physical activity in relation to BMI_SDS_ categories. Participants aged less than 14 years were more likely to have higher BMI compared to other age categories, *p* = 0.035. Students enrolled in private schools exhibited higher BMI compared to those enrolled in public schools (18.5% vs. 13.7%), *p* = 0.017. The frequency of vegetable consumption (*p* < 0.001) and intake of pastries or sweets (*p* = 0.040) were significantly associated with obesity. However, no significant differences in BMI were obtained for the frequency of fruits, soft drinks, fast food, and potato chips consumption (*p* > 0.05). Similarly, differences in parental education level and employment status, income, family size, house ownership, and having a housemaid or driver were not significantly associated with BMI (*p* > 0.05).

### 3.5. Multivariate Logistic Regression Analysis to Determine Predictors of Overweight/Obesity

Multivariate binary logistic regression analysis revealed that participants aged over 16 years were less likely to have overweight/obesity compared to those aged <14 years (aOR = 0.53; 95% CI: 0.35–0.79, *p* = 0.002). Participants enrolled in private schools were at higher risk of overweight/obesity compared to those enrolled in public schools (aOR = 1.55; 95% CI: 1.16–2.08, *p* = 0.003). Participants with daily vegetable intake were less likely than those who never consumed vegetables to be overweight or obese (aOR = 0.47; 95% CI: 0.29–0.77, *p* = 0.002 (Table 6). All the other variables were not significantly associated with BMI_SDS_ after controlling for confounding variables.

## 4. Discussion

Considering the serious burden of obesity at both the national and international levels, the current study was designed to explore the influence of sociodemographic characteristics, dietary habits, physical activity, and daily screen time on BMI_SDS_ values among female adolescent students in Jeddah, Saudi Arabia. In our work, BMI_SDS_ displayed a positive correlation with the dietary habit score, whereas no significant correlation was observed with the sociodemographic score or the physical activity and screen time score.

The results showed that 21.6% and 15.8% of the participants were overweight and obese, respectively. Our results are in line with those reported in previous studies in Saudi Arabia involving female adolescents in both Jeddah and Tabuk cities [16,37], and in other studies involving boys and girls in Riyadh [38,39]. However, some other studies described even higher rates of overweight and obese children in other Saudi cities [15,40,41]. These female obesity rates are higher than those in other Western countries as the USA [7] and Albania [42]. The higher prevalence of obesity in Saudi Arabia is due to rapid urbanization and economic growth witnessed by the Kingdom in the last decades, which have led to significant shifts in lifestyle and dietary habits. Traditional, nutrient-rich diets have increasingly been replaced by energy-dense, processed foods high in fat, sugar, and refined carbohydrates [11,43].

In our work, we observed higher BMI_SDS_ values for participants aged over 16 years and lower BMI_SDS_ values for those younger than 14 years. These results were also confirmed by the multivariate logistic regression. These lower obesity rates among older girls could be explained by better nutritional awareness acquired while growing, which allowed them to make healthier food choices, leading to improved dietary habits and potential decreases in BMI. Also, during early adolescence, girls may experience increases in body fat due to hormonal changes. As they mature, hormonal levels stabilize, and their height increases, which may decrease their BMI in later adolescence [44]. Our findings are in line with a study conducted in Riyadh, where older female adolescents had lower BMI compared to their younger counterparts [45]. They also showed that about two-thirds (69.8%) of adolescents reported that they were not satisfied with their weight. The study attributed the lower BMI in older adolescents to their desire to have an ideal weight, which relates to their image. In fact, as girls progress through adolescence, they often experience heightened body dissatisfaction, leading to increased engagement in weight control behaviors such as dieting and exercise [46]. Another study focusing on female university students aged 18 to 24 years in southwestern Saudi Arabia found a high prevalence of underweight among this group, which was attributed to the increased nutritional awareness, societal pressures regarding body image, and lifestyle modifications [47].

Furthermore, our results showed that the prevalence of being overweight and obese was significantly more pronounced among participants attending private schools rather than public schools. A previous study involving 2906 Saudi adolescents also indicated that those attending private schools exhibited a higher likelihood of being overweight or obese compared with those attending public schools [11]. Although adolescents who attend private schools usually come from families with higher incomes, we did not observe any relationship between BMI_SDS_ and income status or between BMI_SDS_ and the physical activity and screen time score (which was considerably higher for participants with monthly household incomes higher than 14,000 SAR). Because the physical activity and screen time score was not found to be correlated with BMI_SDS_ in our study, we cannot suggest that the relationship between public/private schools and overweight/obese status could be explained by higher income alone. Moreover, physical activity and screen time variables were not significant predictors of BMI_SDS_ when separately assessed using multivariate logistic regression, suggesting further investigation using more comprehensive tools such as the International Physical Activity Questionnaire (IPAQ) [48,49]. On the other hand, previous studies that explored the difference in food choices in public and private schools reported varying results; one study involving schoolgirls aged 12–19 years observed that those attending public schools exhibited healthier dietary habits [50], whereas another involving 1149 children found no difference in intake of selected foods [51]. These differing results imply that the observed difference in overweight/obese status between public versus private schoolgirls warrants further exploration, specifically in terms of elucidating any differences in the food provided by the canteens in the corresponding schools.

The results of this study also indicated that there are no significant associations between the other sociodemographic factors and participants’ BMI_SDS_. Although previous studies revealed higher BMI values with low maternal educational level [52,53,54,55], family income, and having a small family in the house [56], other studies reported, however, no associations between these sociodemographic variables and BMI [57,58].

Multivariate logistic regression revealed a negative association between the frequency of consuming vegetables and BMI_SDS_ (Table 6). These findings are in line with previous reports. A large cross-sectional study involving 13,451 adolescents in southeast Sweden found that a high-frequency intake of vegetables was significantly associated with lower odds of overweight and obesity [59]. Additionally, two meta-analyses reported an inverse relationship between vegetable consumption and BMI among various populations, including adolescents [60,61]. Although the univariate analysis indicated that adolescents who frequently consume pastries and sweets were more likely to be overweight or obese than their counterparts (Table 5), the regression model showed that this association was not statistically significant after adjusting for other variables. It seems that the effect of this variable may be mediated or confounded by other interrelated variables, such as diet or age, when assessed simultaneously in a multivariable context. For the frequency of other food consumption, they were not significantly associated with BMI_SDS_. Other studies conducted in the Eastern and Riyadh provinces of Saudi Arabia also found no significant association between obesity status and the frequency of specific food consumption [57,62]. This may be explained by the fact that the dietary habits information obtained from the participants focused only on the frequency of food consumption but not the portion sizes of foods consumed, which may not have been accurately reflected in their questionnaire responses. Moreover, overweight and obese individuals are more likely to misreport their food intake frequency compared with individuals of normal weight, or consciously avoid unhealthy foods due to weight issues [34]. Similarly, another source of bias could be the self-reported intake frequencies of healthy versus unhealthy foods. For instance, in our study, most of the participants reported consuming fruits and vegetables 2–4 days per week, ≤1 day per week, or never. However, these frequencies were also reported for the consumption of soft drinks, fast food, potato chips, and pastries or sweets.

Finally, a large majority of our study cohort (70.4% of participants) reported engaging in physical activity either never or only ≤1 day per week, while 61.4% reported having >4 h of screen time per day. Altogether, this resulted in 84.9% of participants displaying low or moderate physical activity and screen time scores. High rates of inactivity have also been observed in other studies conducted in Saudi Arabia. One study involving young university students found that 70% of participants reported spending 3–4 h per day watching television, using the internet, or playing Sony PlayStation games [63]. Another study involving female adolescents attending public intermediate and high schools in Jeddah also described an overall low mean physical activity level [37]. These high rates of physical inactivity and sedentary life reported by previous studies may also be explained by the hot and humid climate of Jeddah, high pollution, unavailability of green space, as well as urban density, which are considered important hurdles to outdoor physical activity, especially among female adolescents. This environmental factor may push towards higher levels of sedentary behavior and reduced individual/group outdoor physical activities [64]. Despite the well-known positive health effects of physical activity, as well as the detrimental effects of excess screen time, not all studies have concluded that these lifestyle factors contribute to weight status. For example, one study involving adolescent male students in Saudi Arabia found that the prevalence of overweight and obesity was significantly associated with performing exercise up to and more than three times per week, while watching television did not exhibit a significant relationship with BMI status [15]. In another study, no effect of screen time on BMI status was observed among Saudi adolescents, while those performing moderately or highly vigorous physical activity also displayed a lower probability of being overweight or obese [11]. In our study, the physical activity and screen time scores were not associated with BMI_SDS_ status. Other recent studies reported similar findings, where no association between the frequency of physical activity and BMI was reported [62,65]. This observation warrants further investigation, potentially putting the focus on more specific questions such as the type of physical activity or the amount of physical activity per week (in terms of time, as opposed to frequency). In addition, it should be noted that participants who reported not having a housemaid exhibited higher physical activity and screen time scores, which suggests that they were more involved in household chores and thus more physically active. Furthermore, participants whose fathers had a higher level of education exhibited higher physical activity and screen time scores, which is in agreement with previous studies. A higher level of paternal education may also reflect better knowledge regarding healthier lifestyle habits and the importance of performing regular physical activity.

Among the strengths of the current study is the use of a representative and large sample set of adolescents from both public and private schools in Jeddah. Moreover, the weights and heights used in this study were measured rather than self-reported. This study also includes the use of BMI_SDS_ as a standardized and age-appropriate outcome measure suitable for adolescents, as well as the incorporation of multiple relevant predictors. However, the possibility of bias during the self-reporting of lifestyle choices cannot be ruled out. Additionally, the dietary intake measure mainly focuses on the frequency of consumption of selected unhealthy food items, which limits the understanding of the participants’ overall dietary habits. The questionnaire does not account for meal regularity (e.g., breakfast, lunch, dinner) or a broader range of food items and groups, limiting a comprehensive assessment of the dietary patterns. Similarly, the questionnaire collected information about the frequency of physical activity but not its type or duration. Finally, some participant responses did not align with the predefined answer options and were therefore unclassified, which may have led to minor discrepancies in variable totals and limited the inclusion of those responses in descriptive analysis.

Therefore, further studies need to incorporate a more comprehensive dietary assessment questionnaire that captures a wider range of food items to supply a better understanding of the dietary habits of adolescents. Moreover, using detailed assessment tools for physical activity is needed to achieve more accurate results.

## 5. Conclusions

Overweight and obesity are among the most common public health problems worldwide and affect an increasing number of children and adolescents. The present study examined the associations between several lifestyle and sociodemographic factors and the prevalence of overweight or obesity among female adolescent students in Jeddah, Saudi Arabia. The main findings of this study include that 37.4% of the participants were overweight or obese, and most of them were physically inactive. Also, a higher BMI_SDS_ was positively associated with a younger age and private school attendance. Among the dietary intake, only the frequency of vegetable consumption was negatively associated with BMI_SDS_. No associations were found with the other sociodemographic, dietary intake and physical activity, and screen time exposure variables. These findings emphasize the need for multi-level interventions that consider age-specific behaviors and the influence of school environments on adolescent health outcomes are required to encourage them to embrace healthier lifestyle choices and overcome the burden of obesity among Saudi adolescent students.

## Figures and Tables

**Table 1:** Characteristics of the study participants (*n* = 920).

Variable	Frequency (*n*)	Percentage (%)
**Age (years)**		
<14	214	23.3
14–16	466	50.7
>16	240	26.1
BMI_SDS_ category *		
Underweight	33	3.6
Normal	543	59.0
Overweight	199	21.6
Obese	145	15.8
**Family medical history**		
No medical history	237	25.8
Yes	683	74.2
Diabetes	568	61.7
Hypertension	360	39.1
Cardiovascular disease	121	13.2
Cancer	88	9.6
Other	39	4.2
**School region**		
North	253	27.5
East	195	21.2
South	198	21.5
West	274	29.8
**School type**		
Public	526	57
Private	394	43
**Living status**		
Both Parents	775	84.2
One of Parents	133	14.5
Others	12	1.3

* The body mass index (BMI) standard deviation score (SDS) categories were defined as follows: underweight (SDS < −2), normal weight (−2 ≤ SDS < +1), overweight (+1 ≤ SDS < +2), and obese (SDS ≥ +2).

**Table 2:** Sociodemographic characteristics of the study participants.

Variable	Frequency (*n*)		Percentage (%)	
**Family size** (*n* = 920)				
1–4	125		13.6	
5–7	602		65.4	
˃7	193		21.0	
**House ownership** (*n* = 920)				
Owned	614		67.1	
Rented	303		32.9	
Others	3		0.3	
**Housemaid** (*n* = 920)				
Yes	461		50.1	
No	459		49.9	
**Driver** (*n* = 920)				
Yes	469		51.0	
No	451		49.0	
**Monthly parental income** (*n* = 920)				
<7000 SAR	118		12.8	
7000–14,000 SAR	365		39.7	
>14,000 SAR	437		47.5	
**Maternal education** (*n* = 919) **	***919***			
Illiterate	20		2.2	
Primary/secondary	94		10.2	
High school/diploma	243		26.4	
Graduate studies	562		61.2	
**Paternal education** (*n* = 911) **	***911***			
Illiterate	13		1.4	
Primary/secondary	49		5.4	
High school/diploma	194		21.3	
Graduate studies	655		71.9	
**Maternal occupation** (*n* = 919) **	***919***			
Not working	512		55.7	
Government	233		25.4	
Private	94		10.2	
Business owner	30		3.3	
Retired	50		5.4	
**Paternal occupation** (*n* = 910) **	***910***			
Not working	20		2.2	
Government	457		50.2	
Private	212		23.3	
Business owner	93		10.2	
Retired	128		14.1	
**Sociodemographic Score** (total score = 31) *	**Most Deprived**	**Deprived**	**Moderate**	**Affluent**
	6 (0.7)	169 (18.4)	599 (65.1)	146 (15.9)

* The sociodemographic scores were categorized as “most deprived” (scores of 0–7), “deprived” (8–15), “m” (16–23), or “affluent” (24–31). ** Data is missing due to unclassified responses regarding participants’ parents’ education and occupation.

**Table 4:** Physical activity and screen time (*n* = 920).

	Frequency *n* (%)				
**Weekly physical activity (≥60 min)**	Never	≤1 day per week	2–4 days per week	5–6 days per week	Daily
	336 (36.5)	312 (33.9)	138 (15.0)	54 (5.9)	80 (8.7)
Daily screen time (h) *	None	<2	2–4	>4	
	13 (1.4)	120 (13.0)	222 (24.1)	565 (61.4)	
**Physical activity and screen time score** (total score = 7) **	Low	Moderate	Good	High	
	484 (52.6)	297 (32.3)	119 (12.9)	20 (2.2)	

Data are presented as numbers and percentages. * Daily screen time refers to the amount of time spent watching or using a television/cellphone/tablet. ** The physical activity and screen time scores were categorized as “low activity level” (scores of 0–2), “moderate activity level” (3 or 4), “good activity level” (5 or 6), or “high activity level” (7).

**Table 5:** Association of BMI_SDS_ categories with various study variables (*n* = 920): univariate analysis.

Variable		*n*	BMI SDS				
			Underweight	Normal	Overweight	Obese	*p* Value *
**Sociodemographic characteristics**							
**Age group**	Less than 14	214	10 (4.7)	110 (51.4)	50 (23.4)	44 (20.6)	0.035
	14–16	466	12 (2.6)	277 (59.4)	103 (22.1)	74 (15.9)	
	More than 16	240	11 (4.6)	156 (65)	46 (19.2)	27 (11.3)	
**School type**	Public	526	23 (4.4)	328 (62.4)	103 (19.6)	72 (13.7)	0.017
	Private	394	10 (2.5)	215 (54.6)	96 (24.4)	73 (18.5)	
**Family size**	1–4	125	7 (5.6)	73 (58.4)	25 (20)	20 (16)	0.861
	5–7	602	19 (3.2)	360 (59.8)	128 (21.3)	95 (15.8)	
	>7	193	7 (3.6)	110 (57)	46 (23.8)	30 (15.5)	
**House ownership**	Owned	614	22 (3.6)	358 (58.3)	136 (22.1)	98 (16)	0.939
	Rented	303	11 (3.6)	184 (60.7)	62 (20.5)	46 (15.2)	
	Others	3	0 (0)	1 (33.3)	1 (33.3)	1 (33.3)	
**Housemaid**	No	451	20 (4.4)	278 (60.6)	92 (20)	69 (15)	0.353
	Yes	469	13 (2.8)	265 (57.5)	107 (23.2)	76 (16.5)	
**Driver**	No	451	14 (3.1)	278 (61.6)	93 (20.6)	66 (14.6)	0.435
	Yes	469	19 (4.1)	265 (56.5)	106 (22.6)	79 (16.8)	
**Monthly parental income**	<7000 SAR	118	6 (5.1)	63 (53.4)	30 (25.4)	19 (16.1)	0.784
	7000–14,000 SAR	365	13 (3.6)	217 (59.5)	74 (20.3)	61 (16.7)	
	>14,000 SAR	437	14 (3.2)	263 (60.2)	95 (21.7)	65 (14.9)	
**Maternal education**	Illiterate	20	0 (0)	8 (40)	6 (30)	6 (30)	0.806
	Primary	94	3 (3.2)	53 (56.4)	23 (24.5)	15 (16)	
	High school/diploma	243	10 (4.1)	146 (60.1)	54 (22.2)	33 (13.6)	
	Graduate studies	562	20 (3.6)	335 (59.6)	116 (20.6)	91 (16.2)	
**Paternal education**	Illiterate	13	0 (0)	10 (76.9)	0 (0)	3 (23.1)	0.438
	Primary	49	2 (4.1)	23 (46.9)	11 (22.4)	13 (26.5)	
	High school/diploma	194	8 (4.1)	112 (57.7)	47 (24.2)	27 (13.9)	
	Graduate studies	655	23 (3.5)	391 (59.7)	140 (21.4)	101 (15.4)	
**Maternal occupation**	Housewife	512	20 (3.9)	300 (58.6)	117 (22.9)	75 (14.6)	0.694
	Government	233	7 (3)	147 (63.1)	43 (18.5)	36 (15.5)	
	Private	94	3 (3.2)	46 (48.9)	26 (27.7)	19 (20.2)	
	Business owner	30	1 (3.3)	19 (63.3)	5 (16.7)	5 (16.7)	
	Retired	50	2 (4)	30 (60)	8 (16)	10 (20)	
**Paternal occupation**	Not working	20	1 (5)	13 (65)	4 (20)	2 (10)	0.177
	Government	457	19 (4.2)	276 (60.4)	101 (22.1)	61 (13.3)	
	Private	212	8 (3.8)	115 (54.2)	47 (22.2)	42 (19.8)	
	Business owner	93	0 (0)	48 (51.6)	23 (24.7)	22 (23.7)	
	Retired	128	5 (3.9)	83 (64.8)	23 (18)	17 (13.3)	
**Dietary intake**							
**Fruits**	Never	91	5 (5.5)	55 (60.4)	21 (23.1)	10 (11)	0.148
	≤1 day per week	391	10 (2.6)	251 (64.2)	75 (19.2)	55 (14.1)	
	2–4 days per week	264	10 (3.8)	149 (56.4)	61 (23.1)	44 (16.7)	
	5–6 days per week	54	3 (5.6)	32 (59.3)	9 (16.7)	10 (18.5)	
	Daily	120	5 (4.2)	56 (46.7)	33 (27.5)	26 (21.7)	
**Vegetables**	Never	101	6 (5.9)	61 (60.4)	14 (13.9)	20 (19.8)	<0.001
	≤1 day per week	247	13 (5.3)	146 (59.1)	65 (26.3)	23 (9.3)	
	2–4 days per week	237	4 (1.7)	139 (58.6)	53 (22.4)	41 (17.3)	
	5–6 days per week	101	4 (4)	67 (66.3)	7 (6.9)	23 (22.8)	
	Daily	234	6 (2.6)	130 (55.6)	60 (25.6)	38 (16.2)	
**Soft drinks**	Never	235	10 (4.3)	153 (65.1)	47 (20)	25 (10.6)	
	≤1 day per week	405	13 (3.2)	224 (55.3)	97 (24)	71 (17.5)	0.196
	2–4 days per week	166	7 (4.2)	90 (54.2)	36 (21.7)	33 (19.9)	
	5–6 days per week	53	2 (3.8)	35 (66)	7 (13.2)	9 (17)	
	Daily	61	1 (1.6)	41 (67.2)	12 (19.7)	7 (11.5)	
**Fast food**	Never	32	1 (3.1)	19 (59.4)	5 (15.6)	7 (21.9)	0.056
	≤1 day per week	437	14 (3.2)	243 (55.6)	104 (23.8)	76 (17.4)	
	2–4 days per week	318	12 (3.8)	183 (57.5)	74 (23.3)	49 (15.4)	
	5–6 days per week	82	3 (3.7)	63 (76.8)	7 (8.5)	9 (11)	
	Daily	51	3 (5.9)	35 (68.6)	9 (17.6)	4 (7.8)	
**Potato chips**	Never	92	2 (2.2)	54 (58.7)	19 (20.7)	17 (18.5)	0.712
	≤1 day per week	427	14 (3.3)	249 (58.3)	96 (22.5)	68 (15.9)	
	2–4 days per week	273	12 (4.4)	156 (57.1)	62 (22.7)	43 (15.8)	
	5–6 days per week	77	1 (1.3)	51 (66.2)	15 (19.5)	10 (13)	
	Daily	51	4 (7.8)	33 (64.7)	7 (13.7)	7 (13.7)	
**Pastries or sweets**	Never	38	1 (2.6)	18 (47.4)	11 (28.9)	8 (21.1)	0.040
	≤1 day per week	390	9 (2.3)	217 (55.6)	86 (22.1)	78 (20)	
	2–4 days per week	314	16 (5.1)	191 (60.8)	65 (20.7)	42 (13.4)	
	5–6 days per week	75	1 (1.3)	49 (65.3)	16 (21.3)	9 (12)	
	Daily	103	6 (5.8)	68 (66)	21 (20.4)	8 (7.8)	
**Daily number of meals (including snacks)**	<3	443	14 (3.2)	256 (57.8)	100 (22.6)	73 (16.5)	0.559
	3–5	410	17 (4.1)	240 (58.5)	90 (22)	63 (15.4)	
	>5	67	2 (3)	47 (70.1)	9 (13.4)	9 (13.4)	
**Physical activity and screen time**							
**Weekly physical activity (≥60 min)**	Never	336	17 (5.1)	205 (61)	67 (19.9)	47 (14)	0.347
	≤1 day per week	312	10 (3.2)	171 (54.8)	81 (26)	50 (16)	
	2–4 days per week	138	3 (2.2)	83 (60.1)	25 (18.1)	27 (19.6)	
	5–6 days per week	54	2 (3.7)	36 (66.7)	10 (18.5)	6 (11.1)	
	Daily	80	1 (1.3)	48 (60)	16 (20)	15 (18.8)	
**Daily screen time (h)**	None	13	0 (0)	6 (46.2)	4 (30.8)	3 (23.1)	0.909
	<2	120	3 (2.5)	69 (57.5)	30 (25)	18 (15)	
	2–4	222	8 (3.6)	136 (61.3)	42 (18.9)	36 (16.2)	
	>4	565	22 (3.9)	332 (58.8)	123 (21.8)	88 (15.6)	

* The *p*-values indicate the statistical significance of differences in BMI among the various variables; Significant (*p* < 0.05). The BMI SDS categories were defined as follows: underweight (SDS < −2), normal weight (−2 ≤ SDS < +1), overweight (+1 ≤ SDS < +2), and obese (SDS ≥ +2).

**Table 6:** Predictors of overweight/obesity among the participants: Results of multivariate logistic regression analysis (Fixed model).

Independent Variables	B	SE	aOR	95% CI	*p*-Value
Age (years)					
<14 ^a^			1.0		
14–16	−0.250	0.177	0.78	0.55–1.10	0.156
>16	−0.642	0.208	0.53	0.35–0.79	0.002
School type					
Public ^a^			1.0		
Private	0.440	0.150	1.55	1.16–2.08	0.003
Frequency of vegetable intake					
Never ^a^			1.0		
≤1 day per week	−0.466	0.243	0.63	0.39–1.01	0.055
2–4 days per week	−0.313	0.243	0.73	0.45–1.18	0.198
5–6 days per week	−0.436	0.290	0.65	0.37–1.14	0.133
Daily	−0.755	0.249	0.47	0.29–0.77	0.002

^a^: Reference category. B: Slope. SE: Standard error. aOR: Adjusted odds ratio. CI: Confidence interval. Frequency of intake of pastries and sweets and fast foods was removed from the final model (not significant).

## Data Availability

The data that support the findings of this study are available from the corresponding author upon reasonable request.
